# Sinonasal Melioidosis in a Returned Traveller Presenting with Nasal Cellulitis and Sinusitis

**DOI:** 10.1155/2013/920352

**Published:** 2013-07-07

**Authors:** Rebecca Sin Mei Lim, Sam Flatman, Markus C. Dahm

**Affiliations:** Ear, Nose & Throat Department, Southern Health, Monash Medical Centre, 246 Clayton Road, Melbourne, VIC 3168, Australia

## Abstract

We illustrate a case involving a 51-year-old man who presented to a tertiary hospital with sepsis secondary to an abscess of the nasal vestibule and pustular eruptions of the nasal mucosa. Associated cellulitis extended across the face to the eye, and mucosal thickening of the sinuses was seen on computed tomography. The patient underwent incision and drainage and endoscopic sinus surgery. Blood cultures and swabs were positive for a gram-negative bacillus, *Burkholderia pseudomallei*. He had multiple risk factors including travel to an endemic area. The patient received extended antibiotic therapy in keeping with published national guidelines. Melioidosis is caused by *Burkholderia pseudomallei*, found in the soil in Northern Australia and Asia. It is transmitted via cutaneous or inhaled routes, leading to pneumonia, skin or soft tissue abscesses, and genitourinary infections. Risk factors include diabetes, chronic lung disease, and alcohol abuse. It can exist as a latent, active, or reactivated infection. A high mortality rate has been identified in patients with sepsis. Melioidosis is endemic in tropical Northern Australia and northeastern Thailand where it is the most common cause of severe community-acquired sepsis. There is one other report of melioidosis in the literature involving orbital cellulitis and sinusitis.

## 1. Melioidosis of the Nose

### 1.1. Case

A 51-year-old Caucasian man presented to a tertiary hospital in Melbourne, Australia, with 7 days of headache, myalgia, fevers, and sweats. There was cellulitis affecting the right side face with purulent discharge from a tender right nostril. He had poorly controlled type 2 diabetes requiring insulin and chronic hepatitis B. He was a 25-pack-year smoker, drank >6 standard drinks of alcohol per day, and occasionally snorted cocaine. He also travelled multiple times to Vietnam and worked as a cabinet maker.

He was septic (temperature 39°C, heart rate 110 bpm and blood pressure 90/70 mmHg) with cellulitis extending from the nose across the right cheek and up to the right eye, including a preseptal orbital cellulitis. Visual acuity and eye movements were normal. The right nasal vestibule was tender and swollen with purulent discharge originating from it and the nasal septum.

**Figure 1:**
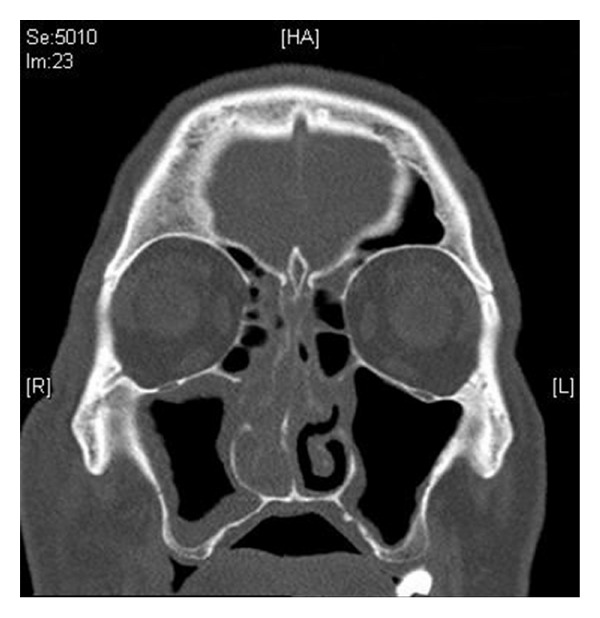
Coronal CT view of sinuses showing right-sided sinusitis with mucosal thickening of the maxillary sinus and anterior ethmoids.

**Figure 2:**
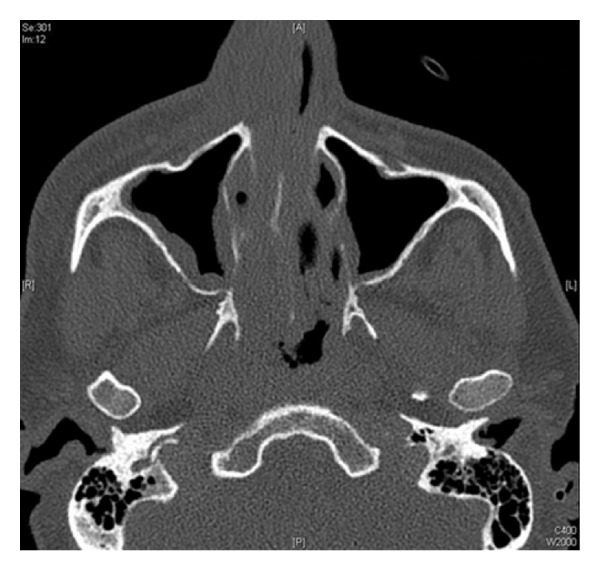
Sagittal CT view of sinuses showing thickening of the mucosa of the anterior right side septum and soft tissue thickening overlying the right side nose and cheek.

Biochemistry revealed an elevated C-reactive protein of 222.7 (*N* < 5) and a normal white cell count of 10.2 × 10^9^/L (*N* = 4.0–11.0 × 10^9^/L), neutrophils 1.95 × 10^9^/L (*N* = 2.00–8.00), and lymphocytes 0.53 × 10^9^/L (*N* = 1.00–4.00). Computed tomography (CT) revealed right sinus mucosal thickening and preseptal tissue swelling overlying the right orbit extending over the zygoma and the nose, with no intracranial collection, cavernous sinus thrombosis, orbital collection, or bony erosion (Figures 1 and 2).

**Figure 3:**
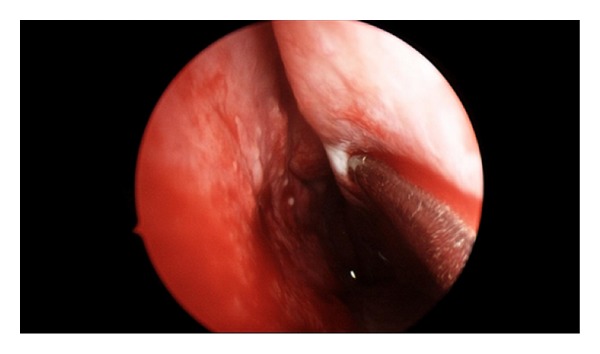
Coalescing pustular eruptions involving the nasal mucosa.

**Figure 4:**
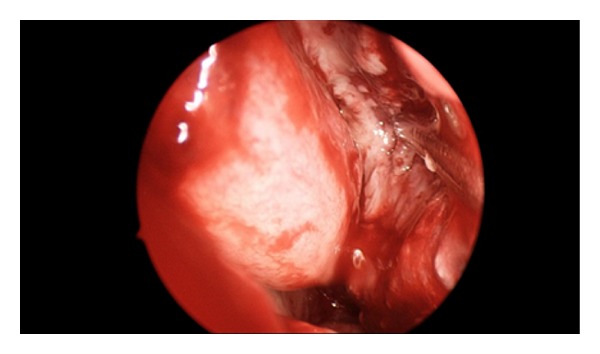
Right nasal vestibule and right septal abscess with boggy, inflamed nasal mucosa.

The patient underwent endoscopic sinus surgery. A right nasal vestibule and septal abscess were identified with boggy, inflamed nasal mucosa (Figures 3 and 4). Incision and drainage were performed and necrotic tissue debrided, with samples sent for histopathology and culture. Septal cartilage was intact. A right uncinectomy, middle meatal antrostomy and anterior ethmoidectomy were performed. No pus was seen in the sinuses.

Blood cultures and swabs from the nasal cavity revealed a gram-negative bacillus, *Burkholderia pseudomallei* (sensitive to ceftazidime, cotrimoxazole, and meropenem). The patient spent 5 days in intensive care, requiring ionotropes for ongoing sepsis, and a further 4 days on the ward. He received daily nose toilet, saline sinus rinse, topical nasal decongestants, strict blood sugar control, and initial treatment with intravenous meropenem. Treatment included a further 8 weeks intravenous ceftazidime 2 g qid as a “hospital in the home” patient, followed by 3 months oral Bactrim DS 2 tabs bd with folic acid 5mg daily and doxycycline 100 mg bd. 

## 2. Discussion

Melioidosis is caused by *Burkholderia pseudomallei*, found in the soil and water of endemic regions, particularly Asia and Northern Australia. It is also known as Whitmore's disease, named after Captain Alfred Whitmore who in 1911 first isolated the bacteria from an opiate addict in Rangoon, Burma [1]. 

The annual incidence in the general northern territory population is 19.6 cases per 100 000 population and increases with wet weather [2].

Transmission of melioidosis is via percutaneous or inhaled routes. The latter was documented during the Vietnam War where American soldiers were reported to have been infected by the aerosol created by helicopters taking off from rice paddies [3].

Acute melioidosis has an incubation period ranging from 1 to 21 days. Animal-to-human and human-to-human transmissions are rare [4]. Isolation of *B. pseudomallei* from bodily fluids of patients remains the gold standard in diagnosis.

The most significant risk factors include diabetes, alcohol abuse, chronic lung, and chronic renal disease [5]. Infected diabetics have a mortality rate of up to 15% [5].

Melioidosis can present with acute, chronic, or latent infection with reactivation. Pneumonia is the most frequent manifestation, followed by genitourinary infections, skin infections, bacteraemia without evident focus, septic arthritis, osteomyelitis, and encephalomyelitis [5–8].

The acute phase of therapy for melioidosis involves at least 14 days of monotherapy with either ceftazidime, meropenem, or imipenem. Doxycycline with or without trimethoprim-sulfamethoxazole is the mainstay of eradication phase therapy, which lasts at least 3 months [9].

The overall mortality rate for melioidosis is about 14%, with the mortality from septic shock as high as 86%. 97.4% of the deceased have at least one risk factor [5].

Our patient was immunosupressed with poorly controlled diabetes, hepatitis B, and alcohol abuse. He was likely originally infected in Vietnam, an endemic area, and had presented a year previously with a necrotising pneumonia diagnosed as melioidosis. However, he failed to comply with treatment and was lost to followup. Reactivation was likely from a pustule or abscess in the nose, leading to extension to surrounding tissues and sepsis. 

A single previous case of orbital cellulitis and sinusitis was described in a 42-year-old previously healthy gentleman from Singapore in 1996 [10].

Melioidosis is a significant opportunistic infection with a high mortality rate in immunocompromised patients. In an age of international travel, surgeons in Australia need to consider melioidosis, especially in a returned traveller from an endemic region.
